# Sex Variations in the Oral Microbiomes of Youths with Severe Periodontitis

**DOI:** 10.1155/2021/8124593

**Published:** 2021-10-20

**Authors:** Ya-Qiong Zhao, Ying-Hui Zhou, Jie Zhao, Yao Feng, Zheng-Rong Gao, Qin Ye, Qiong Liu, Yun Chen, Shao-Hui Zhang, Li Tan, Marie Aimee Dusenge, Jing Hu, Yun-Zhi Feng, Fei Yan, Yue Guo

**Affiliations:** ^1^Department of Stomatology, The Second Xiangya Hospital of Central South University, Changsha 410011, Hunan, China; ^2^National Clinical Research Center for Metabolic Diseases, Hunan Provincial Key Laboratory of Metabolic Bone Diseases, and Department of Metabolism and Endocrinology, The Second Xiangya Hospital of Central South University, Changsha 410011, Hunan, China; ^3^Hunan Key Laboratory of Oral Health Research & Hunan 3D Printing Engineering Research Center of Oral Care & Hunan Clinical Research Center of Oral Major Diseases and Oral Health & Xiangya Stomatological Hospital & Xiangya School of Stomatology, Central South University, Changsha 410008, China

## Abstract

**Objective:**

Periodontitis is an inflammatory disease of microbial etiology caused primarily by dysbiosis of the oral microbiota. Our aim was to compare variations in the composition of the oral microbiomes of youths with severe periodontitis according to gender.

**Methods:**

Subgingival plaque samples collected from 17 patients with severe periodontitis (11 males and 6 females) were split for 16S rRNA gene sequencing. The composition, *α*-diversity, and *β*-diversity of the patients' oral microbiomes were compared between the males and the females. Linear discriminant analysis effect size (LEfSe) was used to analyze the specific taxa enriched in the two groups. Functional profiles (KEGG pathways) were obtained using PICRUSt based on 16S rRNA gene sequencing data.

**Results:**

The Chao1 index and phylogenetic diversity whole tree were significantly higher in males than in females. The Simpson and Shannon indices were not significantly different between the two groups. *β*-Diversity suggested that the samples were reasonably divided into groups. The Kruskal-Wallis test based on the relative abundance of species, combined with the LEfSe analysis showed that the dominant bacteria in males were *Pseudomonas* and *Papillibacter*, whereas the dominant bacteria in women were Fusobacteriales and *Tannerella*. KEGG analysis predicted that the variation in the oral microbiome may be related to the immune system in women, whereas immune system diseases were the dominant pathway in men.

**Conclusion:**

We found sex-specific differences in the oral microbiome in a sample of youths with severe periodontitis. The differences may be related to changes in immune homeostasis and lead to a better understanding of periodontitis.

## 1. Introduction

Disturbances in the oral microbiota can cause an immune response by the host that affects the protection and support of the periodontium, resulting in the development of periodontal disease [1]. Periodontitis is a highly prevalent oral disease among adults, with a prevalence of up to 50% in developed countries [2]. The prevalence is even greater (~90%) in developing countries [3]. The global burden of periodontitis is increasing with life expectancy and due to a worldwide decrease in tooth loss. In the 4th National Oral Health Survey in mainland China, the frequency of adults with periodontitis was 52.8% and with severe periodontitis (stage III or IV) 10.6% [4]. Understanding the composition and structure of oral microbiomes could improve periodontitis prevention, making it important for public health.

Periodontitis is directly preceded by a dynamic, polymicrobial oral microbiome. As a bacterial community develops, the ecological succession from a microbial community to a state of dysbiosis manifests as emergence of newly dominant community members rather than the appearance of novel species [5]. The specific groups and combinations of bacteria, including the “red complex,” *Porphyromonas gingivalis (P. gingivalis)*, *Treponema denticola* (*T. denticola*), and *Tannerella forsythensis (T. forsythensis)*, have been strongly associated with the pathology of periodontitis [6]. These bacteria can alter host immune competence with increased production of virulence factors. For example, the *P. gingivalis* virulence factors have been shown to decrease the host response [7] by subverting innate immune signaling. Though the relationship with the host is normally homeostatic, by manipulating the crosstalk between complement and Toll-like receptors (TLRs) [8], a destructive change is triggered [9]. In addition, the virulence factors of *P. gingivalis* can specifically recognize epitope-specific CD4+ T cell phenotypes [10]. Furthermore, a cluster of species with a less stringent association with disease was defined as the “orange complex” and includes *Prevotella* spp., *Fusobacterium* spp., and *Parvimonas micra* (formerly *Peptostreptococcus micros*) [11]. They were also found to be associated with immunity. *Fusobacterium nucleatum* (*F. nucleatum*) could have the capacity to induce a downregulation of antimicrobial peptides, such as h*β*D-1 and LL-37, but this downregulation of the host defense may be another bacteria-mediated virulence mechanism [12].

Previous studies have suggested that both host genetic and immunological factors are important in oral microbiome dysbiosis, further demonstrating the complex nature of this condition. Race/ethnicity, psychosocial stress, socioeconomic status, gender, and other sociodemographic factors are also gaining more and more importance in the incidence and severity of periodontitis and alterations to oral microbiomes [13]. However, the influence of gender in the process remains controversial. No sex relationship was found for yeasts and staphylococci in a microbiological analysis of 3075 “refractory” periodontitis patients [14]. Similarly, a study that analyzed the relationship of gender and race with components of the subgingival microflora from individuals with different degrees of periodontal disease and periodontal health found no significant differences between males and females [15]. In contrast, Umeda et al. suggested an association between gender and carriage of a specific organism, demonstrating that *Prevotella intermedia/nigrescens* (*P. intermedia/P. nigrescens*) is more likely to be found in the saliva and subgingival and supragingival plaques of males than females [16].

In the current study, we identified sex-specific differences in the oral microbiomes of youths with severe periodontitis through 16S rRNA gene sequencing and predicted the variation of the oral microbiomes that may be related to immunity in order to gain further understanding of periodontitis.

## 2. Materials and Methods

### 2.1. Participants and Inclusion Criteria

We enrolled a total of 17 participants (11 males and 6 females) who visited the medical examination center at The Second Xiangya Hospital of Central South University. The inclusion criteria were patient age between 20 and 44 years; the presence of at least 15 existing natural teeth (excluding third molars); no removable partial denture, bridge, or implant; no antibiotic use within 1 month and no periodontal treatment within the last 6 months; no other bacterial infectious oral disease or systemic disease; and the patient was a nonsmoker. All of the participants received a comprehensive oral examination, which included a professional assessment by a specialized dentist based on the standards of the 2017 World Workshop on the Classification of Periodontal and Peri-Implant Diseases and Conditions “Staging and Grading of Periodontitis: Framework and Proposal of a New Classification and Case Definition” [17]. Before measurement, the sites were air-dried. “Initial” periodontitis was defined as at least one tooth with probing depth (PD) ≥ 3 mm and attachment loss (AL) ≥ 3 mm or PD ≥ 4 mm and AL ≥ 3 mm in ≤30% of teeth. “Moderate” periodontitis was defined as PD ≥ 5 mm and AL ≥ 4 mm in <30% of teeth or PD ≥ 4 mm and AL ≥ 3 mm in 30-60% of teeth. “Severe” periodontitis was PD ≥ 5 mm and AL ≥ 4 mm in ≥30% of teeth or PD ≥ 4 mm and AL ≥ 3 mm in ≥60% of teeth. Patients with severe (stage III) and advanced (stage IV) periodontitis were recruited [18]. The participants were divided into two groups based on gender. The study was approved by the Ethics Committee of The Second Xiangya Hospital of Central South University. All participants were informed of the research aims and provided verbal and written consent. The clinical trial registration number is ChiCTR2100046828.

### 2.2. Subgingival Plaque Collection

Patients were sampled after the oral examination. Supragingival plaque was removed carefully before prior to sampling. The subgingival plaque was collected using a sterile Gracey curette. The curette was introduced into the bottom of the site, and the plaque content was removed in a single stroke into a 1.5 mL tube containing 1 mL of PBS. Samples were stored at -80°C.

### 2.3. DNA Extraction and 16S rRNA Gene Library Preparation and Sequencing

Genomic DNA was isolated from each sample using the DNeasy PowerSoil Kit (QIAGEN) following the manufacturer's instructions. We assessed the yield, purity, and integrity of the DNA using a NanoDrop 2000 spectrometer (Thermo Fisher Scientific Inc., MA USA) and agarose gel electrophoresis as appropriate. 16S rRNA was completed by oebiotech (Shanghai, China). 16S rRNA gene amplification was performed in two steps. First, the V3-V4 hypervariable region was amplified by PCR using the genomic DNA and the following primers: forward, 343 F (5′-TACGGRAGGCAGCAG-3′); reverse, 798 R (5′-AGGGTATCTAATCCT-3′). Amplification was performed in a reaction mixture containing 15 *μ*L of 2x Gflex PCR Buffer, 5 *μ*M primer 343 F, 5 *μ*M primer 798 R, 0.75U Tks Gflex DNA Polymerase (Takara), and 50 ng of template DNA in a total volume of 30 *μ*L/sample. Reactions were run in a PCR thermocycler (BIO-RAD) according to the following cycling program: 5 min of denaturation at 94°C, followed by 26 cycles of 30 s at 94°C (denaturing), 30 s at 55°C (annealing), and 20 s at 72°C (elongation), with a final extension at 72°C for 5 min. The amplified products were checked by 1% agarose gel electrophoresis, purified using AMPure XP beads (Agencourt), and amplified in another round of PCR.

In the second step, sequencing primers and adaptors (1x KAPA HiFi Hotstart ReadyMix, 0.5 *μ*M fusion forward and 0.5 *μ*M fusion reverse primer, and 30 ng Meta-gDNA) were added to 2 *μ*L of the diluted amplicons to a total volume of 50 *μ*L. The PCR was run as described above except with 7 cycles. The Qubit quantification system (Life Technologies) was used for quantification of amplicons according to the manufacturer's instructions. In a single tube, the amplification products were pooled in equimolar amounts and the concentration was determined using the Qubit system. Amplicons were sequenced on the Illumina MiSeq System (Illumina Inc., CA, USA).

### 2.4. Bioinformatic and Statistical Analysis

Fastq files were demultiplexed using MiSeq Controller Software (Illumina Inc.). Vsearch (v. 2.4.2) [19] was used for operational taxonomic unit (OUT) clustering at or above 97%. The taxonomy of the OTUs was assigned, and sequences were aligned according to the RDP classifier Naive Bayesian method [20] and the Silva database. The OTUs were analyzed by phylogenetic methods in the Quantitative Insights into Microbial Ecology (QIIME) software (v.1.9.0). We calculated the *α*-diversity (observed species number, Shannon index, Simpson index, Chao index, PD whole tree index, and Good's coverage index) and *β*-diversity (binary Jaccard, unweighted UniFrac distances, and weighted UniFrac distances) based on the rarefied OTU counts. We used the principal component analysis (PCA) and principal coordinate analysis (PCoA) to plot the similarity or difference in the composition of the sample community. Sequences were used for microbial community metagenome prediction with PICRUSt [21] based on the GreenGenes database [22]. The differential taxa analyses were performed with the linear discriminant analysis effect size (LEfSe) using default parameters (the significance threshold of alpha parameter is set to 0.05, and the logarithmic LDA score cutoff value is set to 2.0). Functional inference was identified using Kyoto Encyclopedia of Gene and Genomes (KEGG) pathways. The Wilcoxon test was used to determine differentially abundant KEGG pathways between the two groups.

## 3. Results

### 3.1. Clinical Characteristics of Participants

The mean ± standard deviation (SD) patient age was 35.73 ± 5.93 and 34.17 ± 7.49 for males and females, respectively. There was no statistically significant difference in age between males and females (*P* > 0.05, Table 1).

### 3.2. Sequence Information

We used Illumina MiSeq and QIIME to analyze the microbiome composition in subgingival plaques from patients with severe and advanced periodontitis. An average of 72,118 (min. 70,569, max. 74,730) clean tags was generated from males and 71,104 (min. 64,237, max. 74,255) clean tags from females. After removing chimeras, the number of analyzed tags decreased to an average of 64,454 (min. 61,201, max. 68,461) in males and 66,097 (min. 60,115, max. 68,779) in females. Using Vsearch (v.2.4.2) software to cluster individual sequences with 97% genetic similarity at the species level, we identified 5181 OTUs among 17 samples, with a minimum of 1171 OTUs in a single sample and a maximum of 1752 OTUs (Table 2).

**Figure 1:**
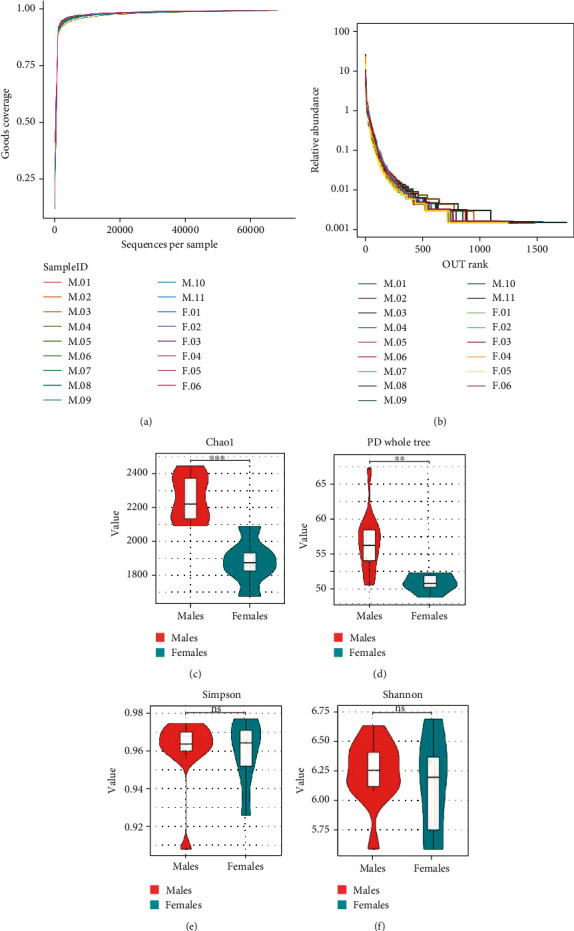
Sequence quality and *α*-diversity analysis of samples from young male and female patients with severe and advanced periodontitis. (a) Rarefaction curves based on Good's coverage for all samples from males and females. The horizontal axis shows the number of operational taxonomic units (OTUs), and appropriate number of sequences is shown on the vertical axis. (b) Rank-abundance curve representative of all samples from males and females. (c–f) Violin plots comparing *α*-diversity indices Chao1, PD whole tree, Simpson index, and Shannon index between the two groups. ^∗∗∗^*P* < 0.001; ^∗∗^*P* < 0.01; ns: not significant.

The quality of sequencing can generally be determined based on the index of the rarefaction curve, rank-abundance curve, or species accumulation curve. In our sample, the rarefaction curves based on Good's coverage tended to be close to saturated, indicating that the number of samples was ample, and there were no obvious differences in the rarefaction curves between the two groups (Figure 1(a)). Similar results were obtained with the rank-abundance curves, which indicate that the amount of sequencing data was abundant enough to reflect most of the microbial species information in the sample (Figure 1(b)).

### 3.3. Diversity Analysis

*α*-Diversity reflects the richness and diversity of oral microbial communities. Bacterial richness and evenness within each group (*α*-diversity) was estimated using the Chao1 index, PD whole tree measurements, and the Simpson and Shannon indices (Figures 1(c)–1(f)). The Chao1 index and PD whole tree were significantly higher in males than females (Wilcoxon rank-sum test, *P* < 0.01). The Simpson and Shannon indices were not significantly different between the two groups (Wilcoxon rank-sum test, *P* > 0.05). Thus, the community diversity was not different in patients with severe and advanced periodontitis according to gender.

**Figure 2:**
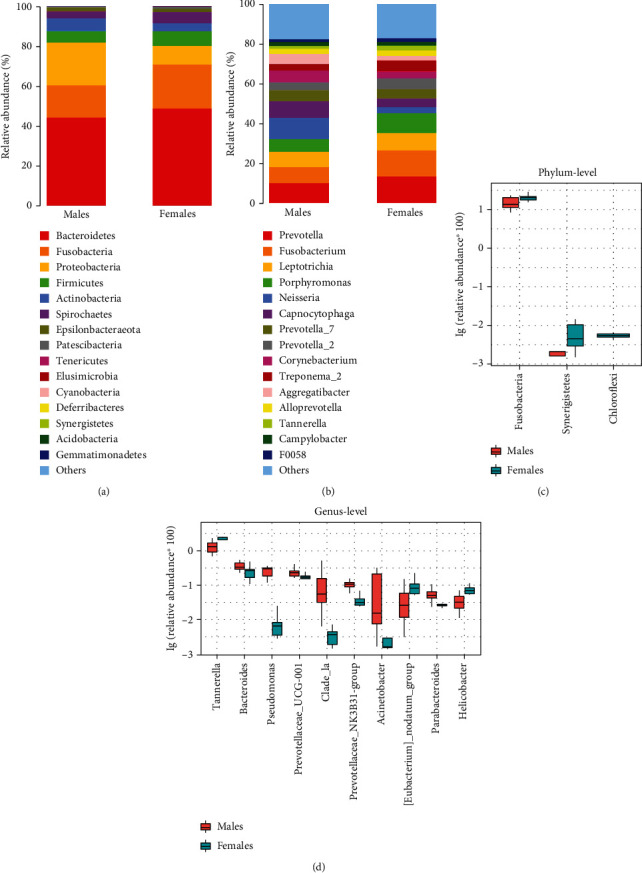
The oral microbiome composition of young male and female patients with severe and advanced periodontitis. (a) Phylum level composition. (b) Genus level composition. Species difference analysis between males and females by the Kruskal-Wallis test at the level of the (c) phylum and (d) genus.

**Figure 3:**
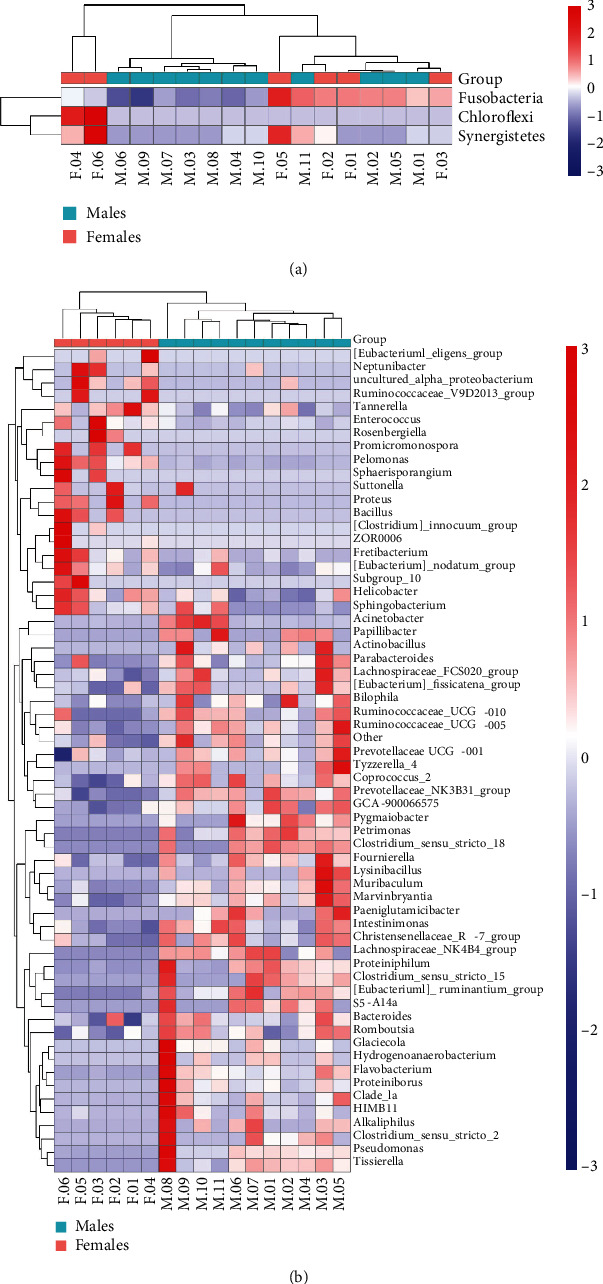
Heat map of the differential oral microbiomes between young male and female patients with severe and advanced periodontitis. (a) Phylum level. (b) Genus level. The horizontal axis is the sample information (group and number), and the vertical axis is the species annotation. Colors indicate the Spearman rank correlation.

Subsequently, for community structure, relative abundance was assessed at the top 15 phylum (Figure 2(a)) and genus (Figure 2(b)) level, respectively. Although the dominant florae were similar, the abundance of each species was vastly different. The main microbiota (>1%) at the phylum and genus level and their abundance are listed in Table 3. Statistical analyses of the major bacteria between the two groups were carried out, and boxplots representing the abundance of the top 3 different species at the phylum and top 10 different species at the genus levels (*P* < 0.05) are shown in Figures 2(c) and 2(d). Compared to males, the relative abundance of *Fusobacteria*, *Synergistetes*, and *Chloroflexi* at the phylum level increased in females (*P* < 0.05), in which the abundance of *Chloroflexi* in males is 0 (Figure 2(c)). At the genus level, the relative abundance of *Tannerella*, *Eubacterium_nodatum_group*, and *Helicobacter* was higher in females than in males (*P* < 0.05) while the relative abundance of *Bacteroides*, *Pseudomonas*, *Prevotellaceae_UCG-001*, *Clade_la*, *Prevotellaceae_NK3B31-group*, *Acinetobacter*, and *Parabacteroides* in females was lower than that in males (*P* < 0.05; Figure 2(d)).  Figure 3 shows the Wilcoxon analysis of differences between males and females in the relative proportions at the phylum (Figure 3(a)) and genus (Figure 3(b)) levels in the form of a heat map.

**Figure 4:**
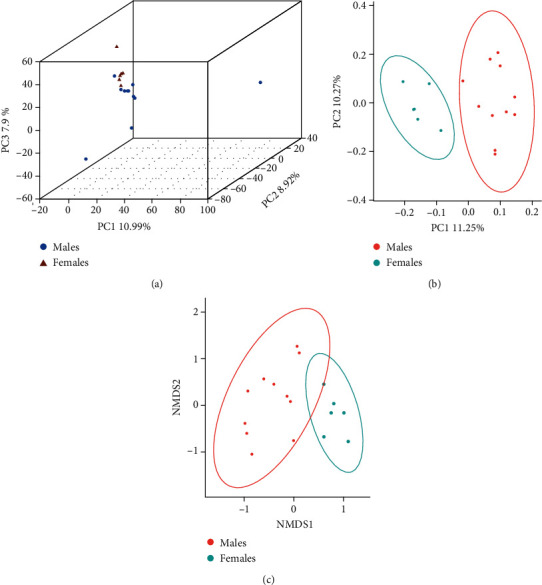
*β*-Diversity analysis of young male and female patients with severe and advanced periodontitis. (a) The similarity of the microbial communities between males and females was analyzed by principal component analysis, (b) principal coordinate analysis, and (c) nonmetric multidimensional scale (NMDS) based on the unweighted UniFrac distance.

*β*-Diversity reflects the differences of microbial diversity between two groups. Therefore, we evaluated the extent of the similarity between the microbial communities using PCA, PCoA, and nonmetric multidimensional scaling (NMDS) analysis based on unweighted UniFrac distances. Males and females with severe and advanced periodontitis could form relative clusters (Figures 4(a)–4(c)) and generally be separated into a two-dimensional spatial distribution, indicating that the two communities were different (Figures 4(b) and 4(c)). An ANOSIM test of the *β*-diversity demonstrated a significant difference (*R* value with the unweighted UniFrac distance matrix = 0.4515; *P* = 0.001).

### 3.4. LEfSe Analysis

**Figure 5:**
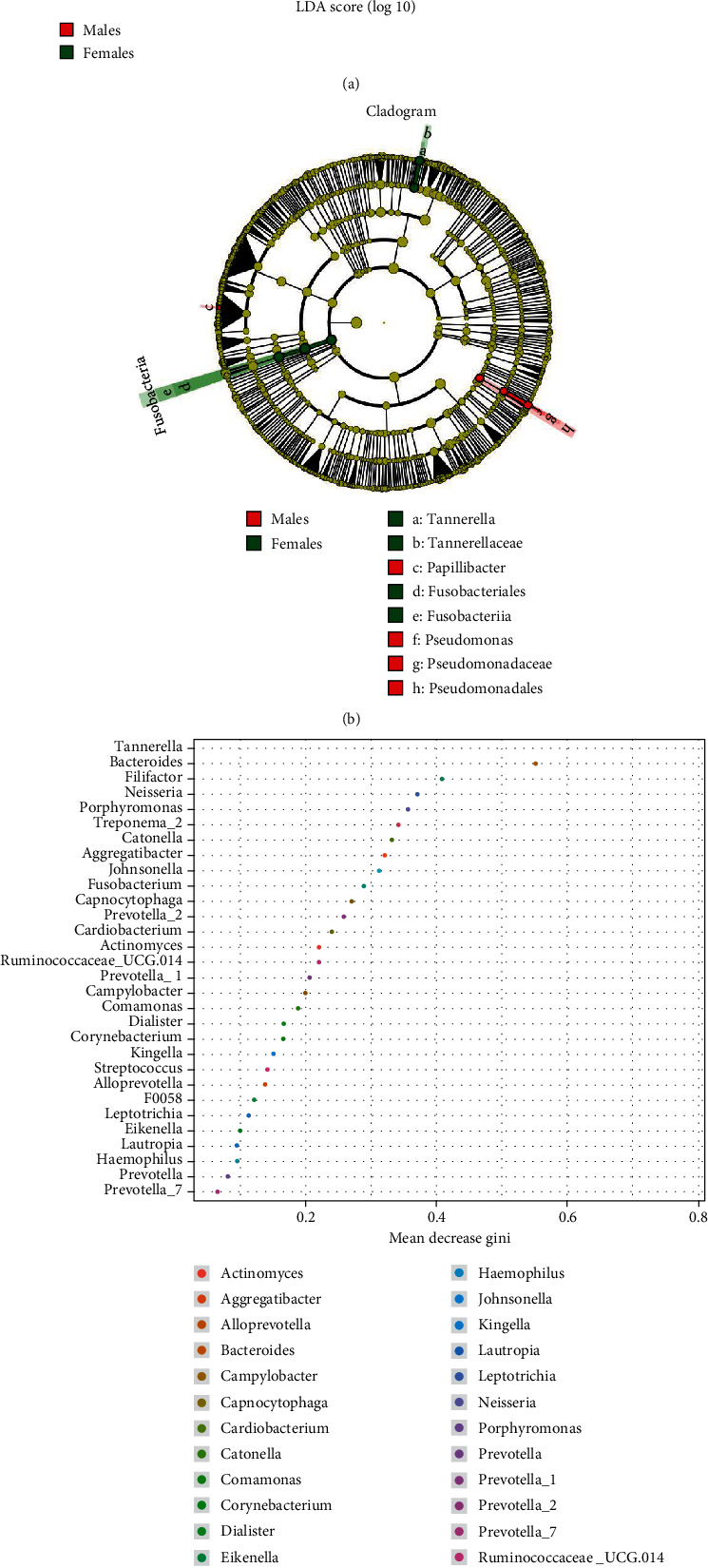
Linear discriminant analysis (LDA) effect size (LEfSe) analysis of the oral microbiomes in young male and female patients with severe and advanced periodontitis. (a) Histogram of the LDA scores of males and females. Female-enriched taxa are indicated by a positive LDA score (green), and taxa enriched in males have a negative score (red). (b) Taxonomic cladogram obtained from LEfSe analysis. Red indicates male, green indicates female, and yellow indicates nonsignificant between males and females. (c) Point map of species importance. The horizontal axis is the measure of importance, and the vertical axis is the name of the species sorted by importance.

The linear discriminant analysis effect size (LEfSe) was used to identify taxa characterizing the differences between the two groups. Based on the results of the species-abundance comparison between the two groups of oral microbiotas, the community differences between groups were analyzed at the phylum to genus level using LEfSe analysis (Figures 5(a) and 5(b)). Our results suggest that the phylum Fusobacteria, its class Fusobacteria, and its order Fusobacteriales, along with the family Tannerellaceae and its genus *Tannerella*, were abundant in the female patients. In addition, the order Pseudomonadales and its family Pseudomonadaceae and its genus *Pseudomonas*, along with the genus *Papillibacter*, were abundant in the male patients. *Tannerella* was the key component that could make the difference between groups by selecting the first 30 genera of relative abundance and carrying out the random forest feature selection procedure (Figure 5(c)).

### 3.5. Functional Metabolism Pathway Prediction

**Figure 6:**
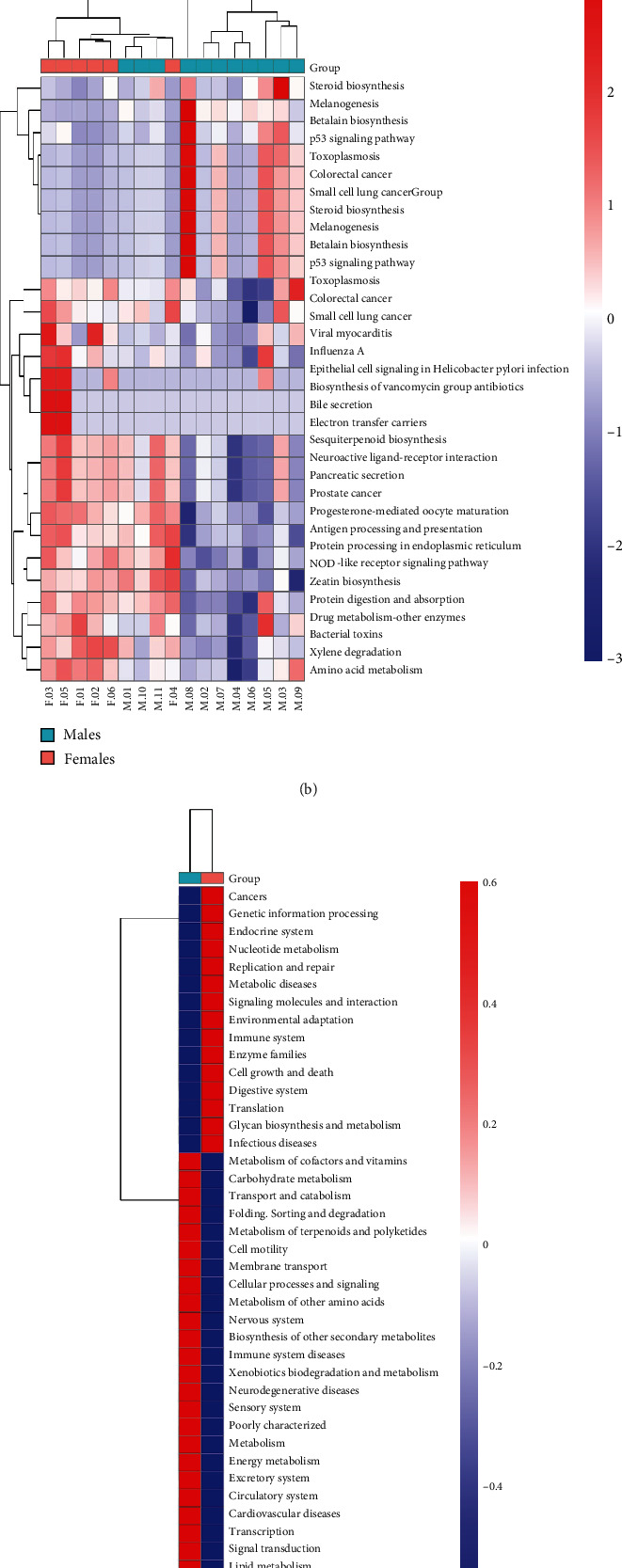
Pathway enrichment analysis based on KEGG. (a) Heat maps of differential pathways at the phylum level and the class level of KEGG in individual patients. (c) Heat map of differential pathways between males and females at the phylum level of KEGG.

Among the KEGG pathways predicted for microbial function, we identified immune-related pathways that exhibit a significant difference in abundance between males and females at different levels in Wilcoxon tests (Figure 6). The pathway analysis of the predicted KEGG pathways in each sample at the phylum level indicated that the microbiomes with altered abundance are mainly involved in pathways related to the immune system and cardiovascular disease (Figure 6(a)). The results at the class level also showed that the pathways were related to the immune system, including the NOD-like receptor signaling pathway and antigen processing and presentation (Figure 6(b)). The predicted KEGG pathways based on group showed that the immune system was also significantly enriched in females and immune system diseases concentrated in males (Figure 6(c)).

## 4. Discussion

In this study, we used 16S rRNA high-throughput sequencing to determine the differences in species abundance in the oral microbiomes of young men and women with severe periodontitis. Regarding *α*-diversity based on species richness, males had higher values than females. In addition, *β*-diversity suggested that the samples were divided into reasonable groups. The Kruskal-Wallis test based on the relative abundance of species composition, combined with LEfSe analysis showed that the dominant bacteria in males were *Pseudomonas* and *Papillibacter*, whereas the dominant bacteria in women were the order Fusobacteriales and the genus *Tannerella*. KEGG analysis predicted that the variation in the oral microbiome may be related to the immune system in women, whereas immune system diseases are the dominant pathway in men, offering further understanding of periodontitis.

Periodontitis is an inflammation that extends deep into tissue and leads to the loss of supporting connective tissue and alveolar bone [23]. Oral microbiomes are defined as the microorganisms found in the oral cavity, including bacteria, viruses, fungi, protozoa, and archaea [24]. The dysbiosis hypothesis states that the transition from periodontal health to disease occurs due to changes in species abundance among the bacteria in the periodontal pocket. This shift in the composition of the microbial community is sufficient to alter the host-microbe crosstalk, resulting in destructive inflammation and bone loss [7]. The healthy oral microbial community usually has low diversity and richness [25]. The species diversity of the general flora is lower in youth than in the elderly, which is often reflected in the periodontal condition of young people being healthier. In addition, young people are more affected by hormones, so there is clinical significance to discussing gender differences. We found that the Chao1 index and PD whole tree were significantly higher in males than females, representing the greater bacterial richness in men, and that they may have severe periodontal tissue destruction. An investigation of the gut microbiota in healthy Japanese subjects found that, although there were significant differences in the microbial structure between males and females aged 20–89 years, the *α*-diversity of the gut microbiota was not different between males and females or among age groups [26].

At the American Academy of Periodontology Workshop held in 1996, experts agreed there are 11 microorganisms that are closely related to periodontal disease, including gram-negative bacteria such as *P. gingivalis*, *T. forsythia*, *P. intermedia/P. nigrescens*, *F. nucleatum*, *Aggregatibacter actinomycetemcomitans* (*A.actinomycetemcomitans*), and *T. denticola* [27]. Among them, we found that *Fusobacteriales* are significantly higher in women than in men through the Kruskal-Wallis test at the level of phylum and LEfSe analysis. The most prevalent in females was *Fusobacteria*, anaerobic gram-negative bacilli that cause tissue necrosis, septicemia, intra-amniotic infections, premature labor, and disorders of the oral cavity, such as pulpal infections, alveolar bone abscesses, and periodontal disease. *F. nucleatum* induces production of inflammatory cytokines and cell proliferation and inhibits apoptosis, cellular invasion, and migration through host cell genomic alterations [12]. *F. nucleatum* reacts to the inflammatory response during periodontal disease and induces secretion of salivary antimicrobial peptides that have an impact on host cells and modulate the immune response [12]. Kostic et al. have indicated that Fusobacteria generates a proinflammatory microenvironment conducive to the progression of colorectal neoplasia by recruiting tumor-infiltrating immune cells [28]. The phylum Proteobacteria was enriched in male patients. Guan et al. have found that high expression is also present in severe bronchiectasis [29].

Our results showed significant variation in the oral microbiomes of the youths of different genders. In our research, at the genus level, the increased *Porphyromonas*, *Prevotella*, and *Tannerella* in the oral microbiomes of females contributed to the periodontal destruction. *P. gingivalis* is the most extensively studied of all major periodontal pathogenic organisms. The virulence factor of *P. gingival* is lipopolysaccharides (LPS), fimbriae, and capsule [30]. *P. gingivalis* LPS and fimbriae stimulation led to the upregulation of TLR2 expression and proinflammatory cytokine production *in vivo* and *in vitro*, resulting in pathogenic inflammatory bone loss [31]. *P. intermedia* and *P. nigrescens* are often isolated from periodontal sites and were once considered to be two different genotypes of *P. intermedia* [32, 33]. *P. intermedia* LPS can participate in bone destruction by stimulating the differentiation and activity of osteoclasts and promoting the release of matrix metalloproteinases from osteoclasts and osteoblasts [34]. It is worth noting that we found that *Tannerella* is significantly increased in women through the LEfSe analysis. *Tannerella* is gram-negative, obligate anaerobic, nonmotile, pleomorphic bacilli [35] that can affect the host immune system through induction of proinflammatory cytokines (e.g., IL-1*β* and IL-6) via CD4+ T helper cells and TNF-*α* [36]. A clinical cross-sectional study found that the presence of *P. gingivalis* and high colonization by *T. denticola* and *P. intermedia* play an important role in severe periodontitis in a Thai population [37]. Besides, the severity of periodontitis in the youths was related to the high level of hormones. In a cohort study of 106 women (50–58 years old), hormone replacement therapy resulted in fewer positive samples for the periodontal pathogens *P. gingivalis*, *P. intermedia*, and *Tannerella forsythia* in the subgingival plaque [38].

Our results show that *Neisseria* and *Capnocytophaga* are increased in men with severe periodontitis, which is consistent with previous results. Minty et al. reported that *Capnocytophaga* were overrepresented in salivary samples from males compared to females [39]. *Capnocytophaga canimorsus* is a gram-negative bacillus with unique virulence factors that enable it to evade the human immune system; it is present in the oral cavities of 22% to 74% of healthy dogs [40]. Leptotrichia, Prevotella_7, and Prevotella_2 showed no significant difference between males and females. Furthermore, no difference was found between males and females regarding the Simpson and Shannon indices, indicating a large number of similar strains in males and females, which is consistent with the results reported by Chen et al. [41]. In contrast to our results, when Belstrøm et al. used microarrays to examine the oral microbiomes of 292 Danish individuals with low levels of dental caries and periodontitis, they found that diet, BMI, age, and sex did not significantly affect microbial abundance, though socioeconomic status affected the oral microbiome profiles [42]. Other possible factors could explain the differences observed in our study, including genetic variations, social factors, chronology of tooth eruption, and hormonal differences, which could affect the composition of the plaque microbiomes [14, 43].

In our study, KEGG analysis found that the immune system, endocrine system, metabolic disease, and infectious disease pathways were significantly changes in women. This may be related to the fact that the female dominant bacteria F. nucleatum had the ability to form a biofilm and coadhere, which can lead to systemic diseases such as urinary tract infection, bacteremia, pericarditis, and otitis media [44, 45]. But the specific mechanism needs to be further studied. This suggests that the imbalance in oral microbiota participates in the regulation of immunological and metabolic homeostasis according to gender differences. However, through the LEfSe analysis, the number of *Papillibacter* was significantly higher in males. Some studies have found that the high relative abundance of *Papillibacter rumminococcacea* is related to melanoma patients with a high response to PD-1 immunotherapy [46], indicating that *Papillibacter* is closely related to immune disease, which is consistent with the enrichment of KEGG analysis in males. Furthermore, *Papillibacter* can cause local inflammation, resulting in aggregation of *α*-synuclein and generation of Lewy bodies that can participate in the occurrence and development of Parkinson's disease in males [47]. This is possibly related to a phenomenon referred to as the mobile microbiome, which is thought to contribute to systemic disease onset and progression. The phenomenon comprises shifts in the oral microbiome; resulting alterations in the local host-immune response and spillage of proinflammatory mediators into the systemic circulation could influence systemic inflammation and immune system diseases [48]. Moreover, nearly all immune cells express the hormone receptors [49, 50], and many immune-related genes possess androgen receptor-responsive elements and estrogen receptor-responsive elements in their promoters, which may be another factor affecting sex differences in the immune responses [51, 52].

## 5. Conclusions

In summary, our study proved the hypothesis of a sex-specific association between the oral microbiomes and severe periodontitis in the youth through 16S rRNA gene sequencing. In addition, KEGG pathway analysis predicted that the variation in the oral microbiome may be related to immune homeostasis. Therefore, further investigation is needed to deepen our understanding of the mechanism.

## Figures and Tables

**Table 1:** Age information of the enrolled participants.

Group	Number	Age
Group 1	11	35.73 ± 5.93
Group 2	6	34.17 ± 7.49
*P*		0.643

**Table 2:** Estimated tags, tag quality, and species diversity of the samples.

Sample ID	Clean tags	Valid tags	Valid percent	OUT counts	Total OTUs	Observed species	Chao1	Good's coverage
M.01	71022	62912	88.58%	1432	5181	1402.3	2363.8	0.989402
M.02	74730	66072	88.41%	1350	5181	1292.7	2087.1	0.990492
M.03	71745	66208	92.28%	1752	5181	1687.9	2435.0	0.989183
M.04	72892	61201	83.96%	1286	5181	1276	2142.8	0.990585
M.05	72755	68461	94.10%	1473	5181	1405.5	2110.3	0.991535
M.06	73836	63441	85.92%	1469	5181	1435	2197.9	0.990143
M.07	70749	63789	90.16%	1452	5181	1416.3	2212.6	0.989977
M.08	72424	65118	89.91%	1380	5181	1330.8	2317.6	0.989657
M.09	71942	65394	90.90%	1510	5181	1457.7	2395.8	0.98916
M.10	70637	62832	88.95%	1544	5181	1512.3	2364.3	0.989155
M.11	70569	63563	90.07%	1462	5181	1425.6	2083.2	0.990467
SM ± SEM	72118 ± 1304	64454 ± 1929	89.39 ± 2.65%	1465 ± 115	5181 ± 0	1422.0 ± 107.6	2246.4 ± 126.3	0.9900 ± 0.0007
F.01	71274	66186	92.86%	1235	5181	1188.4	1831.3	0.99166
F.02	74255	68779	92.63%	1328	5181	1250.9	2082.6	0.990988
F.03	73743	68612	93.04%	1325	5181	1245.7	1934.6	0.99093
F.04	70808	64919	91.68%	1249	5181	1207.8	1909.5	0.991316
F.05	72305	67972	94.01%	1240	5181	1179.6	1817.0	0.991786
F.06	64237	60115	93.58%	1171	5181	1170.6	1672.0	0.99285
SM ± SEM	71104 ± 3307	66097 ± 3006	92.97 ± 0.74%	1258 ± 55	5181 ± 0	1207.2 ± 31.2	1874.5 ± 125.4	0.9916 ± 0.0006

SEM: standard error of the sample mean; SM: sample mean.

**Table 3:** The main microbiota (>1%) at the phylum and genus level and their abundance.

Level	Microbiota	Males	Females
Phylum level	*Bacteroidetes*	44.23%	48.77%
	*Fusobacteria*	15.92%	22.01%
	*Proteobacteria*	21.57%	9.29%
	*Firmicutes*	5.83%	7.36%
	*Actinobacteria*	6.46%	4.07%
	*Spirochaetes*	3.26%	5.56%
	*Epsilonbacteraeota*	1.97%	1.87%
Genus level	*Prevotella*	9.89%	13.29%
	*Fusobacterium*	8.25%	13.16%
	*Leptotrichia*	7.6%	8.78%
	*Porphyromonas*	6.35%	9.87%
	*Neisseria*	10.63%	4.07%
	*Capnocytophaga*	8.31%	4.43%
	*Prevotella_7*	5.62%	4.72%
	*Prevotella_2*	3.99%	5.42%
	*Corynebacteriumand*	5.72%	3.35%
	*Treponema_2*	3.26%	5.56%
	*Aggregatibacter*	5.23%	2.34%
	*Alloprevotella*	2.6%	2.69%
	*Tannerella*	1.37%	2.43%
	*Campylobacter*	1.92%	1.79%
	*F0058*	1.32%	1.79%

## Data Availability

Raw reads have been deposited at NCBI under the BioProject accession number PRJNA763727.
